# Assessment of ADAMTS-13 Level in Hospitalized Children with Serious Bacterial Infections as a Possible Prognostic Marker

**DOI:** 10.3390/medicina55080503

**Published:** 2019-08-20

**Authors:** Linda Rautiainen, Anna Cirko, Jana Pavare, Reinis Balmaks, Ilze Grope, Irina Katirlo, Gita Gersone, Peteris Tretjakovs, Dace Gardovska

**Affiliations:** 1 Lapland Central Hospital, 96400 Rovaniemi, Finland; 2 Department of Paediatrics, Riga Stradins University, LV1007 Riga, Latvia; 3 Department of Human Physiology and Biochemistry, Riga Stradins University, LV1007 Riga, Latvia

**Keywords:** serious bacterial infections, children, sepsis, ADAMTS-13

## Abstract

*Background and objectives*: In children, acute infection is the most common cause of visits in the primary care or emergency department. In 2002, criteria for diagnostics of pediatric sepsis were published, and then revised in 2016 as “life-threatening organ dysfunction due to a dysregulated host response to infection”. In the pathophysiology of sepsis endothelial dysfunction plays a very important role. Deficient proteolysis of von Willebrand factor, due to reduced ADAMTS-13 activity, results in disseminated platelet-rich thrombi in the microcirculation. ADAMTS-13 deficiency has been detected in systemic inflammation. The clinical relevance of ADAMTS-13 during sepsis is still unclear. We aimed to investigate the possible use of ADAMTS-13 as a prognostic marker in children with serious bacterial infection (SBI). *Materials and Methods:* Inclusion criteria were hospitalized children with SBI, aged from 1 month to 17 years. SBI was defined based on available clinical, imaging, and later also on microbiological data. Sepsis was diagnosed using criteria by The International Consensus Conference. In all the patients, the levels of ADAMTS-13 were measured at the time of inclusion. *Results:* Data from 71 patients were analyzed. A total of 47.9% (34) had sepsis, 21.1% (15) were admitted to the ICU, 8.5% (6) had mechanical ventilator support, and 4.2% (3) patients had a positive blood culture. The median level of ADAMTS-13 in this study population was 689.43 ng/mL. Patients with sepsis, patients admitted to the Intensive Care Unit, and patients in need of mechanical ventilator support had significantly lower levels of ADAMTS-13. None of the patients had ADAMTS-13 deficiency. In patients with SBI, the area under the curve (AUC) to predict sepsis was 0.67. A cut-off ADAMTS-13 level of ≤730.49 had 82% sensitivity and 60% specificity for sepsis in patients with SBI. *Conclusions:* ADATMS-13 levels were lower in patients with SBI and sepsis, but AUC and sensitivity were too low to accept it as a prognostic marker.

## 1. Introduction

In children, acute infection is the most common cause of a visit in the primary care or emergency department, and serious bacterial infections (SBI)—due to low incidence, non-specific initial presentation, and the risk of rapid deterioration—make assessing children with acute infection difficult [1,2].

In 2002, The International Consensus Conference on Pediatric Sepsis and Organ Dysfunction produced consensus clinical definitions of systemic inflammatory response syndrome (SIRS) and sepsis in children [3], which became a landmark of diagnostics of pediatric sepsis. However, in 2016, The Third International Consensus Definition Task Force published a definition for sepsis as a “life-threatening organ dysfunction due to a dysregulated host response to infection”. The task force sought clinical criteria for sepsis identification in patients with infection [4]. The criteria have been validated in adults, and in 2017, Travis et al. published an article about the adaptation and validation of these criteria for children [5].

In the pathophysiology of sepsis, endothelial dysfunction plays a very important role as its endothelial activation induces a pro-coagulant state [6]. The von Willebrand factor (vWF) mediates platelet adhesion and aggregation at sites of vascular injury, and is released from the stimulated endothelium as an unusually large (UL) multimer. ULvWF is the hyperactive form of von Willebrand factor (vWF), having more affinity for platelets favoring platelet aggregation; it relies upon an enzyme known as ADAMTS-13 (a disintegrin and metalloprotease with thrombospondin type-1 motif, member 13) for its cleavage and thus conversion into a less active form. Deficient proteolysis of ULvWF due to reduced ADAMTS-13 activity results in disseminated platelet-rich thrombi in the microcirculation [7]. Decreased levels of ADAMTS-13 are particularly seen in thrombotic thrombocytopenia purpura (TTP). Low levels have also been observed in various other disease states including liver disease [8], disseminated intravascular coagulation [9], and severe sepsis [10].

Mild-to-moderate ADAMTS-13 deficiency has been detected in systemic inflammation [11]. Patients with thrombocytopenia-associated multiple organ failure had lower ADAMTS-13 activity, non-survivors also had rich microvascular thromboses on autopsy, and there is data that intensive plasma exchange can replenish ADAMTS-13 activity and reverse organ failure in these children [12].

The clinical relevance of ADAMTS-13 during sepsis is still unclear, although ADAMTS-13 and its role in sepsis have been studied in the pediatric population, where it has been associated with disease severity and outcome [6].

As sepsis is the inflammatory reaction to infection, we aimed to investigate the levels of ADAMTS-13 in children with serious bacterial infection, to detect the possible use of ADAMTS-13 as a prognostic marker for disease severity in children with SBI.

## 2. Materials and Methods

Patient recruitment took place from 1 December, 2014 to 31 December, 2016, in the Children’s Clinical University hospital, Riga, Latvia.

The inclusion criteria were hospitalized children with SBI, aged from 1 month to 17 years. SBI was defined based on the available clinical, imaging, and later also on microbiological data as having either bacteraemia, pneumonia (radiographically confirmed), meningitis, osteomyelitis, intra-abdominal infection, complicated urinary tract infection, skin/soft tissue infection, culture positivity of usually sterile body fluid, or diagnosis by radiology (pneumonia, osteomyelitis, intra-abdominal infection) [13]. Sepsis was diagnosed using criteria by The International Consensus Conference on Pediatric Sepsis and Organ Dysfunction, which was the clinically accepted standard at the time of the study [3].

The exclusion criteria were antibacterial therapy within the last 48 h, immunodeficiency, chronic liver or kidney illness, vaccination within 5 days before the start of the illness, congenital metabolic defects, chromosomal anomalies, and the use of corticosteroids or immunosuppressant medications. Other exclusion factors from the study were obesity, diabetes mellitus, chronic inflammatory diseases, such as rheumatoid arthritis, systemic lupus erythematosus, vasculitis, inflammatory bowel disease, heart diseases, renal or liver diseases, or malignancies and other diseases that are known to be associated with significant changes of anti- and pro-inflammatory biomarkers, including surgery or trauma within the preceding 30 days.

Informed consent was obtained from patients’ parents and additionally from the patients themselves, if applicable. The study protocol was approved by the Committee of Ethics of Riga Stradin’s University on the 24.09.2015 (Nr1/24.09.2015). All of the patients had received the standard of care according to hospital guidelines.

In all the patients, the levels of ADAMTS-13 were measured at the time of inclusion (Quantikine ELISA Human ADAMTS-13 Immunoassay test, Catalog Number: DADT130; R&D Systems, Inc., USA.). As standard diagnostic care, all the patients also had their levels of C-reactive protein (CRP), procalcitonin (PCT), and interleukin-6 (IL-6) measured.

Statistical analysis was performed using IBM SPSS 22. Descriptive statistics were done to identify the characteristics of the categorical and continuous variables. Categorical variables are presented as numbers and percentages. The Kolmogorov–Smirnov test was used to check whether the continuous variables followed normal distribution. The data did not follow a normal distribution; thus, the medians and interquartile ranges are presented. To test for differences between the compared groups, the Mann–Whitney test was used. Correlations were determined by calculating Spearman’s coefficient of correlation (rho), because data were not distributed normally. A receiver-operating characteristic (ROC) curve was developed for the biomarker presenting the area under the curve, including the 95% confidence interval (CI). A two-tailed *p* value of <0.05 was statistically significant.

## 3. Results

Seventy-five patients were included in this prospective study. Later, four patients were excluded from the study population, as their blood samples were hemolytic, and thus not suitable for the analysis of ADAMTS-13 levels. The baseline characteristics of the study sample are depicted in Table 1.

Most cases of SBI were due to pneumonia—78.9% (56); other groups of infections were urinary tract infections 8.4% (6), peritonitis 4.3% (3), bacteremia without a focus 2.8% (2), meningitis 2.8% (2), and osteomyelitis 2.8% (2). The microorganisms that were isolated from the blood cultures in patients with bacteremia were *Streptococcus pneumoniae*, *Haemophilus influenzae*, and *Pseudomonas aeruginosa*. Two patients had positive blood cultures for *Staphylococcus aureus*, which was later ruled to be a contamination. From other cultures, only one patient’s sterile urine sample was positive for *Escherichia coli*. Out of 34 sepsis patients, 5.8% (2) had severe sepsis and 2.9% (1) had septic shock. There was one lethal outcome in this study population.

Levels of ADAMTS-13 were measured at the time of inclusion. The median level of ADAMTS-13 in this study population was 689.43 ng/mL, ranging from 393.68 to 991.58 ng/mL, IQR 583.30–766.16 ng/mL. The analysis of ADAMTS-13 and different disease and treatment factors is depicted in Table 2.

**Figure 1:**
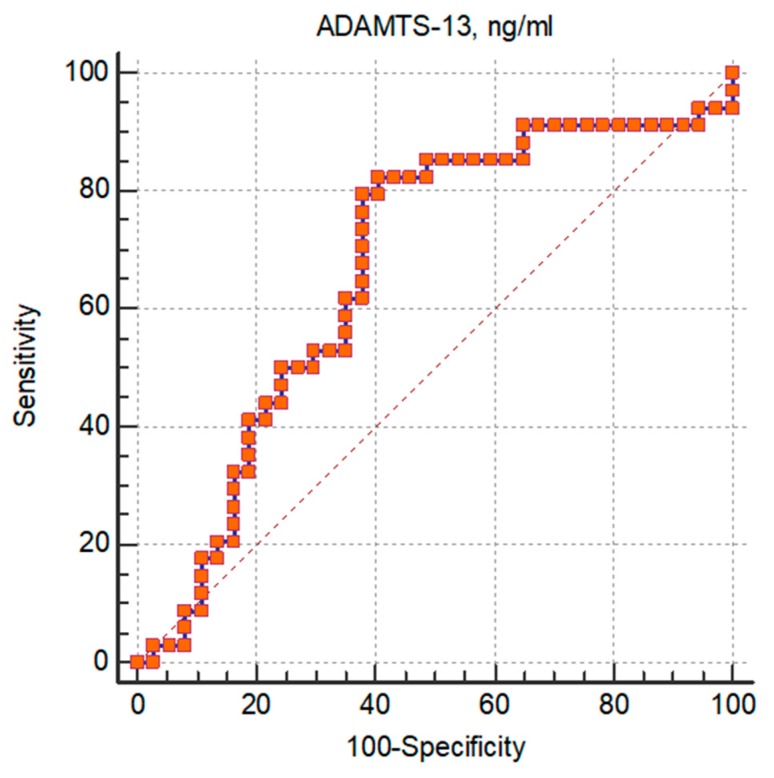
Receiver operator curve (ROC) for ADAMTS-13 in predicting sepsis in study population.

We did not find significant correlations between levels of ADAMTS-13 and other inflammatory markers—CRP (rho = −0.23, *p* = 0.09), PCT (rho = −0.31, *p* = 0.02), and IL-6 (rho = −0.03, *p* = 0.03) respectively. In addition, there was no statistically significant strong correlation between lower ADAMTS-13 level and ICU admission (rho = 0.29, *p* = 0.02), mechanical ventilator support (rho = 0.22, *p* = 0.07), or length of hospitalization (rho = −0.35, *p* < 0.01). Patients with sepsis had significantly lower levels of ADAMTS-13, and its area under the curve (AUC) to predict sepsis in patients with SBI was 0.67 (95% confidence interval (CI) 0.545 to 0.774, *p* = 0.013) (Figure 1). A cut-off ADAMTS-13 level of ≤730.49 had 82% sensitivity and 60% specificity for sepsis in patients with SBI.

As there was only one lethal outcome in the whole study population, statistical analysis of ADAMTS-13 correlation with mortality was not possible.

## 4. Discussion

ADAMTS-13 has been studied recently in adult and pediatric patients with severe sepsis, and it has been shown that patients with severe sepsis have ADAMTS-13 deficiency, and that it may affect in-hospital mortality [7]. Most of these studies focus on ICU patient populations with severe sepsis and septic shock. There have been almost no studies of ADAMTS-13 levels in children with SBI, with or without sepsis.

In a study published in 2015 by Aibar et al., levels of ADAMTS-13 were analyzed in patients with non-infectious SIRS and septic syndromes [14], and the septic patients had lower levels of ADAMTS-13 than the patients with non-infectious SIRS. Our study sample showed a significantly lower level of ADAMTS-13 in patients with sepsis versus patients with SBI without sepsis.

Studies that focus on ADAMTS-13 deficiency and its role in the development of organ failure have set levels lower than 350 ng/mL as a cut-off value [7]. In our study sample the median ADAMTS-13 level was 689.63 ng/mL, ranging from 393.68 to 991.58 ng/mL; thus, none of the patients had ADAMTS-13 deficiency. This is most likely due to the different study population, consisting of pediatric patients with SBI, a relatively low ICU admission rate, only two patients with severe sepsis and one with septic shock, and only one lethal outcome in the whole study group. However, our patients with sepsis, ICU admission, and the need for mechanical ventilatory support had significantly lower levels of ADAMTS-13. Other authors have measured ADAMTS-13 activity in pediatric patients with sepsis and non-sepsis-induced organ failure, and its activity was significantly lower in sepsis-induced-organ failure [15]. Our study sample was too small to statistically analyze ADAMTS-13 differences in patients with or without organ failure; however, we did find a significantly lower mean level of ADAMTS-13 in patients in need of mechanical ventilator support.

Other authors have studied ADAMTS-13 activity in patients with severe sepsis and septic shock and compared those with healthy controls [10]. Whilst ADAMTS-13 activity was lower in patients with severe sepsis and septic shock, they did not find correlations with ADAMTS-13 activity and disease severity, organ dysfunction, or outcome. Other studies as well have confirmed that ADAMTS-13 activity level can be low in sepsis; however, they state that ADAMTS-13 activity is not an independent predictor of the severity of sepsis (if based on SOFA score) or mortality [16]. Our data as well did not show correlations between ADAMTS-13 level and factors that could be interpreted as indicators of disease severity—ICU stay, length of ICU stay, or mechanical ventilatory support.

Qing Zhang et al. have published data about risk stratification in pediatric sepsis patients using ADAMTS-13 as one of the markers; in this study the ADAMTS-13 levels on the day of admission were an independent predictor for a 28-day mortality from sepsis and the cut-off value of 221.58 ng/mL had a 62.6% specificity and 81.3% sensitivity [17]. Their findings suggested that the ADAMTS-13 levels, as well as the vWF/ADAMTS-13 ratio, combined with the clinical SIRS criteria could provide a valuable tool for sepsis mortality prediction. Our patients with sepsis had lower ADAMTS-13 levels, its AUC was 0.67, and a cut-off ADAMTS-13 level of ≤730.49 had 82% sensitivity and 60% specificity for sepsis in the patients with SBI; so we could speculate that a different cut-off level could be used as a diagnostic tool for sepsis in patients with SBI.

This study had several limitations, the small study sample being one of them. At the time when data was gathered, criteria for sepsis diagnosis were made according to the International Pediatric Sepsis Consensus conference. However, lately clinical diagnostic criteria for sepsis have been changing, so results could be altered if different clinical criteria were used.

## 5. Conclusions

Lower levels of ADAMTS-13 were seen in patients with SBI and with sepsis, in patients admitted to the ICU, and in patients that needed mechanical ventilator support. In this study, the AUC and sensitivity were too low to accept as prognostic markers.

## Figures and Tables

**Table 1:** Characteristics of the study sample.

**Baseline Characteristics**	
**Sex, percent(n)**	Boys 60.6% (43) Girls 39.4% (28)
**Age, median (IQR), months**	54.0 (21.5–88.0)
**Day of inclusion after symptom onset, median (IQR), day**	4.0 (2.3–6.0)
**Length of hospitalization, median (IQR), days**	8.0 (5.0–11.8)
**Disease Severity**	
**Sepsis, percent (n)**	47.9% (34)
**ICU admission, percent (n)**	21.1% (15)
**Length of ICU stay, median (IQR), days**	5.0 (3.0–6.0)
**Mechanical ventilator support, percent (n)**	8.5% (6)
**Length mechanical ventilator support, median (IQR), days**	4 (2.5–5.0)
**Blood transfusions, percent (n)**	2.8% (2)
**Laboratory Characteristics**	
**White blood cell count (WBC), median (IQR), ×10^9^/L**	11.89 (9.03–19.81)
**Platelet count (PLT) median (IQR), ×10^12^/L**	260 (213–347)
**C-reactive protein (CRP), median (IQR), mg/L**	49.40 (22.57–158.90)
**Interleukin-6 (IL-6), median (IQR), pg/mL**	37.00 (7.51–58.82)
**Procalcitonin (PCT), median (IQR), pg/mL**	0.64 (0.13–3.58)
**Positive blood culture, percent (n)**	4.2% (3)

**Table 2:** Differences in on ADAMTS-13 depending on disease characteristics and treatment, median (IQR), ng/mL.

ICU Admission		*p* = 0.021
Yes (15)	610.92 (527.64–689.67)	
No (56)	721.43 (599.05–796.25)	
Sepsis		*p* = 0.030
Yes (34)	655.10 (565.47–718.57)	
No (37)	743.29 (648.20–820.67)	
Mechanical ventilator support		*p* = 0.040
Yes (6)	567.91 (514.50–693.38)	
No (65)	706.58 (584.98–788.15)	
